# Supplementary material for: Force Generation on the Hallux Is More Affected by the Ankle Joint Angle than the Lesser Toes: An In Vivo Human Study
Source: Biology (Basel). 2021 Jan 12;10(1):48. doi: 10.3390/biology10010048 (PMC7828001; doi:10.3390/biology10010048)
Supplement: Supplementary file 1 [file biology-10-00048-s001.pdf]

Supplementary Table S1. The mean values of the intra-subject coefficient of variation (CV) of the measurement of the first and second–fifth metatarsophalangeal joints (MTPJs). The CV was calculated by dividing the standard deviation of the repeated measurements by mean value.

	CV (%)											
Ankle position	20° plantar-flexion				neutral				20° dorsiflexion			
Dorsiflexion angle of the MTPJs	0°	15°	30°	45°	0°	15°	30°	45°	0°	15°	30°	45°
First MTPJ	3.3	2.9	4.5	2.7	2.5	2.1	3.1	3.3	2.4	2.1	2.7	2.3
Second–fifth MTPJs	3.7	4.1	3.4	2.7	3.3	2.8	3.3	3.6	2.7	3.6	2.7	3.2

Supplementary Table S2. The values of the standard error of the measurement (SEM) of the first and second–fifth metatarsophalangeal joints (MTPJs). The SEM was calculated by dividing the standard deviation of the measurements by the number of subjects.

	SEM (Nm)											
Ankle position	20° plantar-flexion				neutral				20° dorsiflexion			
Dorsiflexion angle of the MTPJs	0°	15°	30°	45°	0°	15°	30°	45°	0°	15°	30°	45°
First MTPJ	0.5	0.4	0.4	0.4	0.5	0.7	0.6	0.8	0.7	0.8	0.8	0.8
Second–fifth MTPJs	0.4	0.4	0.3	0.2	0.6	0.6	0.5	0.6	0.3	0.3	0.3	0.3
